# Some diseases are described in the literature as sometimes acute, sometimes chronic: How do general practitioners take medical decisions when faced with “fluctuating” conditions? A qualitative study

**DOI:** 10.1371/journal.pone.0323760

**Published:** 2025-06-18

**Authors:** Julien Le Breton, Laura Moscova, Florence Pinsard-Laventure, Etienne Audureau, Émilie Ferrat, Pascal Clerc

**Affiliations:** 1 Univ Paris Est Creteil, Département universitaire de médecine générale, F-94010 Creteil, France; 2 Univ Paris Est Creteil, INSERM, IMRB, CEpiA Team, F-94010 Creteil, France; 3 Société Française de Médecine Générale (SFMG), F-92130 Issy-les-Moulineaux, France; 4 Centre de santé universitaire Salvador Allende, F-93120 La Courneuve, France; 5 Maison de santé pluri-professionnelle, F-77120 Coulommiers, France; 6 Université Versailles Saint Quentin, Département universitaire de médecine générale, F-78180 Montigny-le-Bretonneux, France; 7 Maison de santé pluri-professionnelle du théâtre, F-78190 Trappes, France; 8 Service de Santé Publique, Hôpital Henri-Mondor, F-94010 Creteil, France; 9 Maison de santé pluri-professionnelle universitaire de Saint-Maur-des-Fossés, F-94100 Saint-Maur-des-Fosses, France; 10 Maison de santé pluri-professionnelle Philippe Marze, F-78130 Les Mureaux, France; Centre Hospitalier de Troyes, FRANCE

## Abstract

**Background:**

Although there is a clear distinction between the management of typically acute diseases (e.g., a sore throat) and the management of typically chronic diseases (e.g., diabetes), there is also a ‘fluctuating’ zone that includes many medical conditions with a major impact on the healthcare system and the management of which is usually judged to be unsatisfactory.

**Objective:**

To understand how general practitioners (GPs) identify and manage acute, chronic and ‘fluctuating’ (i.e., subacute or recurrent acute) conditions on a day-to-day basis.

**Methods:**

In a qualitative focus group study, 33 French GPs were invited to discuss their management of typical acute diseases, typical chronic diseases, and ‘fluctuating’ conditions. Data saturation was achieved after five focus groups. Thematic content analyses and matrix analyses (disease management key factors vs. diseases studied) were performed.

**Results:**

The disease management key factors were classified into in seven themes: physician-patient negotiation; complex consultations; greater vigilance for patients with multimorbidity; the absence of standard treatments; patient education over time; inapplicable guidelines; and difficult multidisciplinary coordination. The specific management key factors of ‘fluctuating’ conditions were frequent, erratically scheduled consultations; consultations focused on social relationships, work, and the family; the lack of effective drug treatment in most cases; a break in the patient pathway; a lack of initial medical education about these diseases; and medical guidelines judged to be inappropriate with regard to actual practice.

**Conclusions:**

These results challenge the acute/chronic dichotomy that is still applied to disease management but also highlight possible ways of improving the management of ‘fluctuating’ conditions.

## Background

The prevalence of chronic disease is increasing in France and worldwide [1–2]. Chronic disease is a major public health issue in terms of cost, quality of life, and the organisational structure of healthcare systems [3–4]. France’s healthcare system is still focused on the management of acute disease. The fee-for-service payment models correspond to the management of a single problem, to which the physician responds by prescribing a short course of treatment that supposedly will not be repeated [5]. In contrast, the overall management of patients suffering from a chronic disease requires the development of public health strategies that encompass prevention, screening, multidisciplinary care, long-term follow-up, and patient education. The accumulation of several chronic diseases in a given patient (multimorbidity) increases the likelihood of iatrogenic events [6].

Even though there is a clear distinction between the management of typically acute diseases (sore throat, paronychia, and pyelonephritis, for example,) and the management of typically chronic diseases (arterial hypertension, diabetes, and congestive heart failure, for example), it is not always easy to classify some diseases, which can be described as ‘fluctuating’ conditions: is lower back pain acute, recurring, or chronic? Is this the first episode of cystitis or is it a recurrence? Does the patient have an “anxious” profile, an acute stress reaction, or chronic anxiety that worsens his/her quality of life? In this context of “subacute” or “recurrent acute conditions”, there is uncertainty about the need for long-term care. The general practitioner (GP) is used to dealing with common conditions and applying well-known acute care procedures but is often finds managing recurrent conditions challenging. ‘Fluctuating’ conditions are a daily concern for GPs and have major psychological, emotional, personal, professional and socioeconomic impacts for patients, with poor quality-of-life and functional impairment [7–9]. However, to the best of our knowledge, ‘fluctuating’ conditions *per se* have not previously been studied.

The objective of the present study was to understand how GPs identify and manage these ‘fluctuating’ conditions on a day-to-day basis, in order to identify drivers for improving practice.

## Methods

### Study design

The qualitative focus group approach is particularly well suited to exploratory studies, since it enables the opinions of several GPs to be recorded at the same time. The method leverages group dynamics to explore collective perspectives and refine understanding of specific concepts, such as the characterization of ‘fluctuating’ conditions [10].

### Study population

The present study was performed in France (in the Ile-de-France region and the city of Poitiers) between January and December 2019. The study population comprised both established GPs and locums. Participation was voluntary, and participants were recruited via professional mailing lists until data saturation was achieved. We used purposive sampling to include a diverse range of profiles with regard to age, gender, level of professional experience, area of practice, university teaching or research activities, and learned society membership. While some participants were familiar with the investigators prior to the study, the majority had no prior acquaintance with them. No participants declined to take part in the study.

### Data collection

Informed consent from participants was obtained verbally and recorded at the start of the focus group session. Each focus group session lasted an average of 118 minutes (range: 107–126 minutes). The meetings were organized at the French Society of General Practice (SFMG) in Paris and at a practice in Poitiers, audio-recorded (with the participants’ agreement), and led jointly by a senior researcher (JLB, [MD-PhD, male]) and a junior researcher (MLC, [resident, female]). Field notes were made during the focus groups. The data were then transcribed (JLB and MLC) and analyzed; while the focus group guide was designed to be adaptable, only minor rewording adjustments were made to enhance clarity in subsequent focus groups. The sessions comprised three phases: the participants were invited to discuss a “typical acute disease” (a sore throat, for example), a “typical chronic disease” (diabetes, for example) and then several ‘fluctuating’ conditions that are sometimes described in the literature as being acute and sometimes as being chronic (depression, asthma, and low back pain, for example). The focus group guide was based on a study of practice commissioned by the French Observatory of General Practice [11] and a review of the literature on the key factors of the outpatient management of chronic diseases [12]. The guiding questions were “how do you manage a patient who consults for [disease name]?”, “which obstacles to managing [disease name]?”, and “what would you need to better understand [disease name]?”. The question “why are you are not following up patients who have consulted for [disease name]?” was specifically asked only to participants who reported not following up with such patients. The ‘fluctuating’ diseases were selected based on their prevalence in general practice [11], drawing on the clinical experience of the researchers (working in peer groups of the French Society of General Medicine, SFMG) and validated by a review of the literature, particularly focusing on the acute/chronic oscillation described in the existing studies. The list of conditions discussed in the focus groups included: chronic obstructive pulmonary disease (COPD), hyperthyroidism, depression, lower back pain, obesity, alcohol use, migraine, gastroesophageal reflux (GER), cataracts, and psoriasis.

### Analysis

After each focus group session, the data were transcribed and the verbatim was analyzed and coded by two researchers (JLB and PC [MD-PhD, male]), using NVIVO 11 software (QSR International, Melbourne, Australia). In coding reliability thematic analysis, the primary coder (JLB) developed a code list on the basis of the themes that emerged during the review of the first transcript. This code list was reviewed and revised by the secondary coder (PC). Codes were added and revised, and definitions were clarified and differentiated if needed. After the process had been repeated with a second transcript, the two researchers believed that the code list captured the issues raised by respondents. The last three transcripts were then coded by the primary coder using the established framework, with continuous attention to identifying any new codes. Any additional codes that emerged were discussed and validated through discussions among the three researchers before being incorporated into the final analysis

The analysis was carried out within an inductive and constructivist framework, emphasizing flexibility and a participant-centered approach. Rather than being predefined, the themes emerged organically from the data, reflecting the participants’ experiences and meanings [13]. We analyzed these themes to identify key factors in GP-led disease management regardless of the condition’s acute, chronic or ‘fluctuating’ nature. The key factors were defined as a combination of a management approaches, obstacles, and facilitators. Participants were asked to provide feedback on the findings. Those who responded offered insights that helped refine the thematic descriptions and ensured the findings were representative of their experiences. Any significant new perspectives were discussed among the research team and, where relevant, integrated into the final analysis. The quotes were subsequently translated into the English language for this manuscript.

We next performed a matrix analysis of the disease management key factors and the diseases covered in the focus group sessions. A matrix involves the comparison of two categories of data, to see how they interact [14]. We thus sought to identify the key factors that were specific for acute diseases (i.e., those not found for chronic and ‘fluctuating’ conditions), specific for chronic diseases, and specific for ‘fluctuating’ conditions. Certain key factors could not be attributed to one type of disease because they were found for all categories.

Ethical approval was received from the French Society of General Practice’s ethics committee (reference: 04112014). All participants gave informed consent verbally and recorded at the start of the focus group session. All data were kept securely and confidentially in line with ethical requirements, and where data is presented below, all quotes are anonymised to protect participants’ identities.

## Results

A total of 33 GPs participated in the study and formed five focus groups of six to eight individuals. The characteristics of the participating GPs are summarized in Table 1.

**Table 1:** Characteristics of the GPs who participated in the study.

Characteristics of the GPs (n = 33)	N (%)
**Age** (years), median [range]	49 [28; 71]
**Sex** (women)	11 (33.3)
**Years of general practice**, median [range]	18 [0; 45]
**Type of practice**	
* Single-GP practice*	7 (21.2)
* Multi-GP practice*	13 (39.4)
* Multidisciplinary health centre*	13 (39.4)
**Area of practice**	
* Urban area*	31 (93.9)
* Rural area*	2 (6.1)
**Teaching activity**	
* Trainee GP supervisor*	22 (66.7)
* Lecturer*	17 (51.5)
* None*	7 (21.2)
**Research activity**	
* Investigator*	14 (42.4)
* Researcher*	10 (30.3)
* None*	15 (45.5)

### Thematic analysis

The thematic analysis highlighted seven major themes in disease management by GPs: physician-patient negotiation, consultation complexity, risk management, treatment strategies, follow-up planning, guidelines, and healthcare system use. The coding tree is described in Table 2.

**Table 2:** The coding tree, showing the major themes and key factors of GP-led disease management.

Major themes		Key factors of GP-led disease management
**Physician-patient negotiation**	**Approaches**	Negotiation with the patient Shared GP-patient decision-making The patient is a stakeholder in his/her health or disease
	**Obstacles**	A patient who imposes an unnecessary consultation and dictates its content Mismatch between the patient’s and the GP’s expectations Lack of understanding and agreement by the patient An insufficiently committed patient, passive patient Poor adherence The GP imposes constraints on the patient An insufficiently committed GP
	**Facilitators**	Impact on quality of life (comfort) Social consequences for work and family
**Consultation complexity**	**Approaches**	Easy management, a short consultation A short care sequence – a short time interval between two consultations
	**Obstacles**	A weight on the GP – burdensome care Difficult management and a long consultation Complex biomedical and psychosocial management Complex medical management
	**Facilitators**	Frequent consultations
**Risk management**	**Approaches**	Anticipating and preventing risk Risk forecast as a function of the background Rule out a medical emergency or an acute risk Ruling out a serious disease
	**Obstacles**	Complications of a chronic disease
	**Facilitators**	Prescription of additional examinations No risk or low-risk
**Treatment strategies**	**Approaches**	Non-pharmacological treatment Prevention of relapses, patient education Medication review Complementary and alternative medicine
	**Obstacles**	The absence of effective treatments Difficult-to-balance treatments
	**Facilitators**	A switch from short-term treatment to long-term treatment Repeated symptomatic treatment Self-medication
**Follow-up planning**	**Approaches**	Care plan, care schedules, and multidisciplinary working Long-term patient follow-up and support
	**Facilitators**	Rapid reassessment of the patient is possible The role of patient education
**Guidelines**	**Approaches**	Application of guidelines Adaptation of and deviation from the guidelines
	**Obstacles**	Criticism of initial medical education Unclear guidelines Absence of benchmarks, complex care Poor knowledge of the guidelines
	**Facilitators**	The value of guidelines
**Healthcare system use**	**Approaches**	Effective GP-specialist collaboration The role of paramedical staff
	**Obstacles**	Negative impact of the specialist Break in the patient pathway

### The matrix analysis

In the matrix analysis, we cross-checked the management key factors identified in the thematic analysis against the diseases assessed (Table 3).

**Table 3:** Key factors of GP-led disease management, by disease category (acute, chronic or ‘fluctuating’) in a matrix analysis.

	Key factors of GP-led disease management
**Acute diseases**	A short care sequence – a short time interval between two consultations Prescription of additional examinations No risk or low-risk Risk forecast as a function of the background Ruling out a serious disease The role of patient education Rapid reassessment of the patient is possible
**Chronic diseases**	The GP imposes constraints on the patient The patient is a stakeholder in his/her health or disease Poor adherence Shared GP-patient decision-making A weight on the GP – burdensome care A long consultation Complex biomedical and psychosocial management Complex medical management Anticipating and preventing risk Medication review Care plan, care schedules, and multidisciplinary working Long-term patient follow-up and support The value of guidelines Application of guidelines Unclear guidelines Adaptation of and deviation from the guidelines Effective GP-specialist collaboration The role of paramedical staff
**‘Fluctuating’ conditions**	Social consequences for work and family Lack of understanding and agreement by the patient No negotiation with the patient Difficult management and a long consultation Frequent consultations No need to rule out a medical emergency or acute risks No complications of a chronic disease The absence of effective treatments Repeated symptomatic treatment Prevention of relapses, patient education Non-pharmacological treatment Difficult-to-balance treatments A switch from short-term treatment to long-term treatment Self-medication Absence of benchmarks, complex care Poor knowledge of the guidelines Criticism of initial medical education Negative impact of the specialist Breaks in the patient pathway
**Not specific for a type of disease**	Impact on quality of life (comfort) A patient who imposes an unnecessary consultation and dictates its content Mismatch between the patient’s and GP’s expectations An insufficiently committed, passive patient GP with a passive approach Complementary and alternative medicine

#### Acute conditions.

For diseases characterized as “acute”, we found seven key factors (Table 3). The GPs’ response during the consultation was immediate and clinically based. These consultations were simple and short, and more than one consultation was required rarely.

“*On the whole, it’s a consultation that (as mentioned by the other participants) we expect to be quick because the patient is there for a single reason.*” (GP9)

*“You don’t have the impression that he [the patient] is hiding something, that something out of the ordinary is going to happen.*” (GP10)

Patients were not systematically re-examined; any follow-up consultations were quick, with a short duration of care and a brief time interval between visits.

“*We tell the patient to come back if the symptoms are still there after a given period.”* (GP25)

Although GPs generally considered these conditions to be low-risk or predictable based on context and patient history.

“*It is fairly well defined: we’re not really taking a risk.*” (GP15)

“*Paronychia… it all depends on the site… the stage, and the person’s underlying conditions! If she/he has a diabetes or another underlying chronic disease, the person is going to decompensate and get … some type … of skin …infection.”* (GP16)

They occasionally prescribed additional diagnostic assessments to better assess the risk.

“*Prescribe a comprehensive set of tests, and yes, do a detailed ultrasound examination… to find out progressively what’s going on.*” (GP17).

They also emphasized the need to rule out a medical emergency or a serious underlying condition.

“*Indeed, there is always the emergency situation, emergencies with serious, sudden aetiologies.*” (GP13)

*“The sore throat is not the same as usual, it’s not “pure”, and I say to myself, that’s strange, maybe it’s cancer.”* (GP11)

“*But an acute episode is a decompensation (above all in patients with heart problems), we’re always on the look-out for it.”* (GP4)

#### Chronic conditions.

For diseases characterized as “chronic”, we found 18 key factors (Table 3). These consultations were long and complex, requiring both biomedical and psychosocial skills, a prevention-focused approach, follow-up planning, and a medication review. Patients with chronic conditions and multimorbidity were perceived as presenting biomedical or psychosocial challenges, often leading to longer consultations. GPs described these consultations as burdensome.

“*A consultation for an acute disease in a patient with congestive heart failure is probably much more complicated; with renal colic in heart failure, the medications will be a real hassle.*” (GP12)

“*We get straight into something where we have to ask ourselves questions, about the representation of the patient… we are already dealing with things that are more… more complex.”* (GP2)

“*It is quite complicated, it is very time-consuming*” (GP9)

“*Because it is also psychologically complicated for us, you know”* (GP9).

Some patients were passive and insufficiently involved, leading to poor adherence.

“*Except that he’s not a stakeholder in his treatment because he’s still smoking, he’s not very involved.*” (GP5)

“*it’s not us who decides whether he adheres to the treatment*” (GP5)

However, patients were not only seen in this light; they were also portrayed more positively as a stakeholder in his/her health or disease.

“*The patient is a stakeholder in his or her treatment and care, and so we can’t do it without them and we can’t do it without adapting to their rhythm.*” (GP3)

GPs still viewed themselves as needing to impose certain restrictions on patients.

“*The monitoring will have to be more… rigorous, in as much as you’ll have the effects of the treatment too; it’s true that monitoring is sometimes imposed.*” (GP6)

GPs emphasized the importance of negotiation, efforts to improve patient understanding and commitment, and the role of shared decision-making.

“*A* *disease, it’s always a negotiation with the patient*.” (GP7)

“*I sometimes ask them to reformulate [things]: what will you take home from this discussion?*” (GP8)

“*In that field, treatment decisions are truly shared.*” (GP1).

They focused on prevention and risk forecasting.

“*It’s about preventing a life-threating situation. You hope that your patient is not going to have another infarction (if he/she has already had one), that the diabetic patient isn’t going to have one, perhaps it’s more likely to happen, et cetera, et cetera… You’re doing prevention, in fact.*” (GP14)

The complexity of treating patients with chronic disease and multimorbidity meant that GPs needed to spend time reviewing patient’s prescriptions.

“*The follow-up for a chronic disease, well, you have to think about repeat prescriptions, you have to concentrate, it means that you have to consider a lot of things and check that there aren’t too many drug interactions or that it won’t worsen the other diseases.*” (GP9)

For some diseases, GPs mentioned performing long-term follow-up, though they did not necessarily use the term “chronic”.

*“There is a whole set of things to be set up and it’ll last a long time, and it will be… with a view to a long-term, complex treatment.”* (GP22)

They affirmed that they planned the follow-up and drew up a care plan in collaboration with the patient. This follow-up was considered the best approach to educating the patient.

“*You can take it step by step, so that the patient takes a number of things onboard, erm, … and … erm… you set up the follow-up, the rules…*” (GP2)

“*It is really a very specific relationship with each patient, a relationship with explanations, support, and help – a relationship that I think is very meaningful.”* (GP23)

“*Let’s say when they come to renew a prescription and that, for example, they don’t have any other problems, well, I like to try to discuss the diet problem, the weight problem, … it’s doing what one could call patient education … I try but it’s not easy.*” (GP24)

Medical guidelines were generally viewed positively by GPs. While they were often critical of guidelines and standard protocols, they recognized that these tools could be helpful in certain situations.

“*I find that in this disease, it’s all really codified… we’re somewhat systematic.*” (GP7)

It was possible to apply a guideline even when it did not match the reality of the GPs’ practice, as long as it had been adapted accordingly.

“*Because these are deviations from the protocol or adaptations of the protocol but there’s still a general guideline that I often modify very extensively.”* (GP23)

“*But that’s what bothers me: we don’t deal with the patient, we deal with the diabetes.*” (GP25)

The care team included nurses and other paramedical staff, primarily responsible in day-to-day care provision, while specialist physicians were considered by GPs as collaborative partners. In the context of chronic disease, working with specialists and regularly coordinating with nursing staff was the norm.

“*For these patients, I really work quite easily with the cardiologist, as a duo.”* (GP6)

“*We sometimes end up… working with the nurse.*” (GP26)

#### Fluctuating conditions.

For diseases characterized as ‘fluctuating’, we found 19 key factors (Table 3). The GPs resorted to specific types of care: the consultations were frequent, sometimes erratic, and focused on the disease’s impact on the patient’s social relationships, work, and family.

“*Because, in general, we see them quite often – not once every 10 years…*” (GP11)

“*It’s the handicap experienced by the patient; in fact, the level of handicap experienced by the patient determines whether you’ll be more “adventurous” with regard to the treatments or care envisaged.”* (GP1)

“*Someone who is taking a lot* of *[analgesics], with an impact on his/her work, family life and stuff… it’s a completely different situation in terms of the disease burden and chronicity.*” (GP2)

The GPs highlighted the lack of effective treatments and often prescribed non-pharmacological approaches for recurrent conditions, typically as part of a patient education strategy and/or to prevent relapses. Occasionally, they renewed prescriptions for symptomatic medications.

“*Certain physicians (…) stop themselves from saying that there’s a long-term treatment because it is so poorly effective.”* (GP18)

“*For a recurrent condition and a response that is fairly... standard…, we send the patient to the physiotherapist, and in the meantime he’ll go and see the osteopath…*” (GP19)

“*And this also raises the patient’s awareness; it’s patient education, to try to help them give up smoking.*” (GP3)

“*Because I don’t think that there are any long-term treatments for psoriasis; we treat the flare-up when it happens.”* (GP20).

The main challenges were linked to insufficient information about these diseases in the GPs’ initial medical training, and medical guidelines that were often considered poorly aligned with actual practice.

“*It seems so stupid to us as a guideline that... that..., we sit on it!”* (GP5)

The GPs felt that the patient pathway had failed, leading to over-medicalization, iatrogenesis and/or a breakdown in care due to inappropriate referrals to specialists and the excessive prescription of additional examinations and tests.

“*The risk and the chronic nature [of the disease] come from being treated by a rheumatologist…*” (GP19)

“*But when he is referred for psychotherapy, you don’t see him for three years! I’ve got patients that I used to see very regularly, and bingo!*” (GP25)

We also identified six management key factors that were not specific to a single “acute”, “chronic” or ‘fluctuating’ disease category (Table 3). These key factors belonged to two themes only: physician-patient negotiation and the absence of standard treatments. For physician-patient negotiation, the key factors were the impact on quality of life, a mismatch between the GP’s expectations and those of the patient, with (sometimes) an insufficiently committed physician, and/or a consultation imposed by the patient.

“*My problem is just that; the difference between care on a day-to-day basis and the patient’s expectations, which is often, erm, a cure as if by magic!*” (GP3)

“*I consider that it’s* [a] *disease when my patient tells me that she/he is ill with it.”* (GP4)

For the “absence of standard treatments” theme, the key factors included the difficultly balancing effectiveness and safety, deciding whether to switch to a long-term treatment, the use of complementary and alternative medicines, and self-medication.

“*The long-term treatment depends on the patient’s level of discomfort… and in this case, the degree of handicap caused by the migraine; then you decide to initiate long-term treatment or not.*” (GP1).

“*There’s also the thought that there are easily available over-the-counter drugs at the chemists, for self-medication....*” (GP21)

## Discussion

The present study evidenced management key factors that characterized the GP’s medical activity. Diseases were managed during consultations, with seven challenges for medical decision-making: physician-patient negotiation, complex consultations, greater vigilance for multimorbidity patients, the absence of standard treatments, patient education over time, inapplicable guidelines, and difficult multidisciplinary coordination. Our results also highlighted the challenges and ambiguities that GPs face when dealing with conditions that range from acute to chronic, which makes care provision more difficult.

The results confirm that GPs have a key role in the management of recurrent acute conditions [15], with consultations that are frequent, sometimes erratic, and particularly centred on the impact on social relationships, work, and the family. Examples of these recurrent conditions can be found in the literature: recurrent respiratory tract infections in children [15,16], recurrent urinary tract infections in women [17], acute coronary disease [18], low back pain [19], and recurrent depression [20]. The published studies focused on the aetiology, risk factors for recurrence, and therapeutic strategies.

The literature data suggest that preventive measures and the management of recurrences are of value: detailed assessments, an individual prevention plan, early care, good compliance by the patient, and regular follow-up. In our study, GPs used management tools typically associated with either “acute” or “chronic” conditions, depending on how the condition is perceived; this constitutes a risk because the tools might not be fully appropriate. In the literature, therapeutic approaches that systematically help patients manage their conditions over the long term (symptomatic, supportive, specific and/or preventive treatments, and patient education) appear to be more effective than those based on acute care [21–24]. However, most of the studies published to date do not offer a consensus on the effectiveness of prolonged or preventive treatment in reducing recurrent disease. This results in significant variability in the therapeutic strategy.

The GPs in our study pointed out the absence of effective drug treatments for most of these ‘fluctuating’ conditions and expressed concern about the frequent breaks in the patient pathway. Lastly, the GPs highlighted the lack of information about ‘fluctuating’ conditions in their initial medical training and stated that medical guidelines were often poorly suited to actual practice. These data provide a better understanding of the causes and consequences of variability in practice – a grey area for GPs.

Our study highlights the challenges GPs face in managing conditions that do not fit neatly into acute or chronic categories. This echoes the literature on medically unexplained symptoms (MUS), where diagnostic uncertainty and variable symptom patterns complicate care. Like MUS, “fluctuating” conditions require a patient-centered approach and continuity of care [25,26]. Studies on MUS suggest that patient-GP communication, continuity of care, and the integration of biopsychosocial approaches are key factors in improving patient outcomes. However, unlike MUS, they often involve identifiable pathological components, necessitating a nuanced balance between biomedical, psychological, and social management strategies [27,28].

### Strengths and limitations

To the best of our knowledge, the present study is the first to have explored the management of these ‘fluctuating’ conditions by GPs. A large number of GPs participated in the study, which produced in-depth debates that often continued beyond the study’s boundaries. The GPs enjoyed being able to discuss these frequently encountered, day-to-day issues with fellow physicians. The participants’ level of commitment to the focus groups (which lasted for two hours, on average) testified to the significant level of interest in this approach. By promoting dynamic interactions, the focus groups enabled us to explore individual ideas and capture opinions, thanks to interactions between the GPs. We were thus able to record and analyze shared meanings and to take account of disagreements, which would not have been not possible in individual interviews. The use of multiple data analysis methods increased the validity of our analysis and the credibility, stability and reliability of the interpretations elaborated by the researchers. The matrix analysis was decisive and enabled us to explore and clarify the three types of clinical situation: acute, chronic and ‘fluctuating’ conditions. Our study also had a few limitations. Iterative coding was primarily conducted for the first two transcripts, after which the finalized code list was applied to the remaining transcripts. While this approach ensured consistency in coding, it may have limited the identification of new codes that could have emerged in later focus groups. However, discussions among the three researchers helped validate and refine the coding framework throughout the analysis process. During the focus groups, the coding, the verbatim analysis, and our role as “physician-researchers” might have led to over-interpretation of our colleagues’ statements. However, the role also gave us legitimacy for pointing out disagreements, raising hypotheses based on our own experience, and better understanding the participants’ statements [29]. Furthermore, the translation of quotes from French into English might have caused some nuances to be lost.

### Implications for research and/or practice

The characterization of three types of disease – acute, chronic and ‘fluctuating’ – will have consequences for medical decision-making, the organisation of care, and the stakeholder’s remuneration (in multidisciplinary primary care teams, in particular). For diseases characterized as “acute”, the patient-GP relationship is not the key factor; the consultation tends to be driven more by the immediate medical needs, resembling a fairly simple question of supply and demand. However, it is important to acknowledge that consultations for acute issues can still serve as opportunities to build rapport and establish trust. This relational foundation may prove valuable if more complex or long-term concerns arise in the future. Strengthening continuity and communication in these encounters could enhance patient engagement and facilitate better management of ongoing or recurrent conditions [30]. The consultation is over quickly, and the duration is sometimes determined by the patient and not the GP. The medical risk is potentially high, the treatment is essentially drug-based, the follow-up is inexistant or short-term only, and the first-line care team corresponds to the bare minimum (the GP and the community pharmacist).

For diseases characterized as “chronic”, a physician-patient partnership (i.e., a therapeutic alliance) is essential and necessarily involves the GP because it constitutes a long-term, mutual investment. However, this relationship extends to certain specialists consulted frequently, such as the cardiologist, the diabetologist, and the neurologist. The consultation takes a long time, and the biomedical risk (anticipating complications or decompensations as much as possible) has to be managed. The treatment involves polymedication and is complex, with a risk of iatrogenesis. Decisions are shared by the specialist and the patient, in a therapeutic alliance. The follow-up is characterized by a long-term (but regularly revised) care plan. The first-line care team usually comprises the GP, the nurse, the community pharmacist, the specialist physician, and homecare assistants.

For diseases classified as ‘fluctuating’, close relationships and good communication are required for good mutual understanding and commitment. The risk of a mismatch between the patient’s expectations and the GP’s understanding of the situation is high. Patients often seek immediate symptom relief, while GPs may emphasize long-term management strategies, leading to potential frustration and non-adherence. Negotiation is always prominent; a given care sequence must always be followed up by the prescribing physician, who is not necessarily the GP. The consultation is often long and complex, with a moderate medical risk. While some pharmacological options exist for specific conditions, the treatment is essentially non-drug-based, focusing on patient education, self-management support, and lifestyle interventions. This reflects both the lack of curative drug treatments for many “fluctuating” conditions and the recognition that long-term symptom control often requires a multifaceted approach. The dynamic care plan negotiated with the patient calls on a broader first-line care team: the GP, a physiotherapist, a psychologist, social workers, a psychomotor therapist, occupational physicians and physicians working for health insurance funds.

Recurrent acute conditions and chronic diseases have similar consequences: worse quality of life and a psychosocial impact. It is now essential to model the short- and long-term care trajectories of ‘fluctuating’ conditions by analyzing health databases [31] and applying statistical methods that are appropriate for the evaluation of recurrent conditions [32,33].

## Conclusions

Our results showed that there is room for improvement in GPs’ management of ‘fluctuating’ conditions, which do not fit neatly into acute or chronic care models. GPs must navigate diagnostic uncertainty, varying symptom expression, and patient expectations, all while coordinating care across multidisciplinary teams. Although our study does not suggest that GPs systematically mismanage these conditions, it highlights the challenges in aligning treatment strategies with both patient needs and the current healthcare structures. The characterization of this ‘fluctuating’ zone is of critical importance to clinicians, researchers and institutions, as it encompasses a wide range of health problems with significant implications for the healthcare system. Moreover, these conditions are often perceived as being poorly managed by all the stakeholders. A deeper understanding of this category could help improve clinical practice, optimize care coordination, and ultimately enhance patient outcomes.
